# Gene expression of bovine embryos developing at the air-liquid interface on oviductal epithelial cells (ALI-BOEC)

**DOI:** 10.1186/s12958-017-0310-1

**Published:** 2017-11-25

**Authors:** Vera A. van der Weijden, Shuai Chen, Stefan Bauersachs, Susanne E. Ulbrich, Jennifer Schoen

**Affiliations:** 1 ETH Zurich, Animal Physiology, Institute of Agricultural Sciences, Universitätstrasse 2, CH-8092 Zurich, Switzerland; 2 Institute of Reproductive Biology, Leibniz Institute for Farm Animal Biology (FBN), Wilhelm-Stahl-Allee 2, 18196 Dummerstorf, Germany

**Keywords:** *Bos taurus*, Oviduct, Air-liquid interface, Early embryonic development, In vitro embryo production

## Abstract

We recently developed an air-liquid interface long-term culture of differentiated bovine oviductal epithelial cells (ALI-BOEC). This ex vivo oviduct epithelium is capable of supporting embryo development in co-culture up to the blastocyst stage without addition of embryo culture medium. However, blastocyst rates in co-culture were markedly lower than in conventional in vitro embryo production procedures. In the present study, we assessed target gene expression of ALI-BOEC derived embryos to test their similarity to embryos from conventional in vitro embryo culture. We screened previously published data from developing bovine embryos and selected 41 genes which are either differentially expressed during embryo development, or reflect differences between various in vitro culture conditions or in vitro and in vivo embryos. Target gene expression was measured in 8-cell embryos and blastocysts using a 48.48 Dynamic Array™ on a Biomark HD instrument. For comparison with the ALI-BOEC system, we generated embryos by two different standard IVP protocols. The culture conditions lead to differential gene expression in both 8-cell embryos and blastocysts. Across the expression of all target genes the embryos developing on ALI-BOEC did not depart from conventional IVP embryos. These first results prove that gene expression in ALI-BOEC embryos is not largely aberrant. However, there was no clear indication for a more in vivo-like target gene expression of these embryos. This calls for further optimization of the ALI-BOEC system to increase its efficiency both quantitatively and qualitatively.

**Electronic supplementary material:**

The online version of this article (10.1186/s12958-017-0310-1) contains supplementary material, which is available to authorized users.

## Introduction

Establishment and maintenance of a viable embryo and subsequent implantation depends on a complex, dynamic network of endocrine signalling pathways and local factors produced by the embryo and the female reproductive tract [1]. These early embryo-maternal interactions are subtle and difficult to investigate in vivo, especially in monotocous species like cattle. Therefore, reliable in vitro tools faithfully mimicking the early embryonic environment are needed. The simple, columnar-shaped oviduct epithelium builds the first maternal “contact zone” for the early embryo. However, when grown under standard culture conditions, oviduct epithelial cells (OEC) lose their polarized phenotype and dedifferentiate both morphologically and functionally [2, 3]. We recently developed an air-liquid interface (ALI) culture system for the long-term culture of differentiated bovine oviductal epithelial cells (ALI-BOEC) [4]. The ALI-BOEC model forms tissue-like epithelial layers, which actively generate an oviductal fluid surrogate on its apical cell surface. Proteomic analyses revealed the abundance of >3000 proteins with distinct similarities to in vivo oviductal fluid. Initial co-culture experiments with living zygotes have proven that co-cultured embryos develop up to the blastocyst stage. The embryonic development is solely supported by the ALI-BOEC milieu, as no embryo culture medium was supplemented. In the present study, we assessed the stage-dependent gene expression of 41 target genes in embryos produced in this ALI-BOEC system. Selected target genes represented different functional categories (imprinted genes and DNA methylation; stem cell markers and differentiation; apoptosis; embryo development, quality and competence; metabolism). For comparison, we used embryos from two different conventional in vitro embryo production (IVP) protocols. We hypothesized that embryos successfully developing in the environment of the ALI-BOEC system exhibit similar gene expression compared to embryos produced in conventional bovine IVP procedures.

## Methods

### Reagents

Foetal bovine serum, DMEM/Ham’s F12, HEPES, L-glutamine, sodium pyruvate, amphotericin B and penicillin/streptomycin was purchased from Merck Millipore (Billerica, MA, USA). If not otherwise indicated all other reagents were purchased from Sigma Aldrich (St. Louis, MO, USA).

### Animal materials

Bovine ovaries and oviducts were by-products from local slaughterhouses (Teterower Fleisch GmbH, Teterow, Germany; Biopark, Güstrow, Germany). All samples were collected within 15 min after slaughter. Ovaries were maintained at 38.5 °C in pre-warmed DPBS with 1% penicillin, while oviducts were transported to the laboratory on ice.

### In vitro production of embryos

Cumulus oocyte complexes (COCs) were aspirated from 3 to 5 mm follicles. Three different culture conditions were performed: 1) Standard IVP, 2) IVP using a commercial serum-free media suit and 3) Standard IVM/IVF with subsequent co-culture on ALI-BOEC (detailed descriptions see below). The conditions are later referred to as -S (standard), −SF (serum-free), and -ALI (ALI-BOEC co-culture), respectively. For each culture condition two experiments (trial 1 and 2) were conducted to obtain 8-cell embryos and blastocysts, respectively. Each experiment was performed with a distinct pool of oocytes, but frozen-thawed semen from one single proven fertile bull. The cleavage (% cleaved embryos per total number of oocytes) and blastocyst rate (% blastocysts per cleaved embryos) was assessed on day 2 and day 8 of in vitro culture (IVC), respectively. A total of 15 8-cell embryos (randomly divided into 3 groups) and 6 single blastocysts of the same developmental stage (expanded, not hatched) were collected under each culture condition. Embryos were snap frozen in liquid nitrogen and then stored at −70 °C for later processing.

**Fig. 1:**
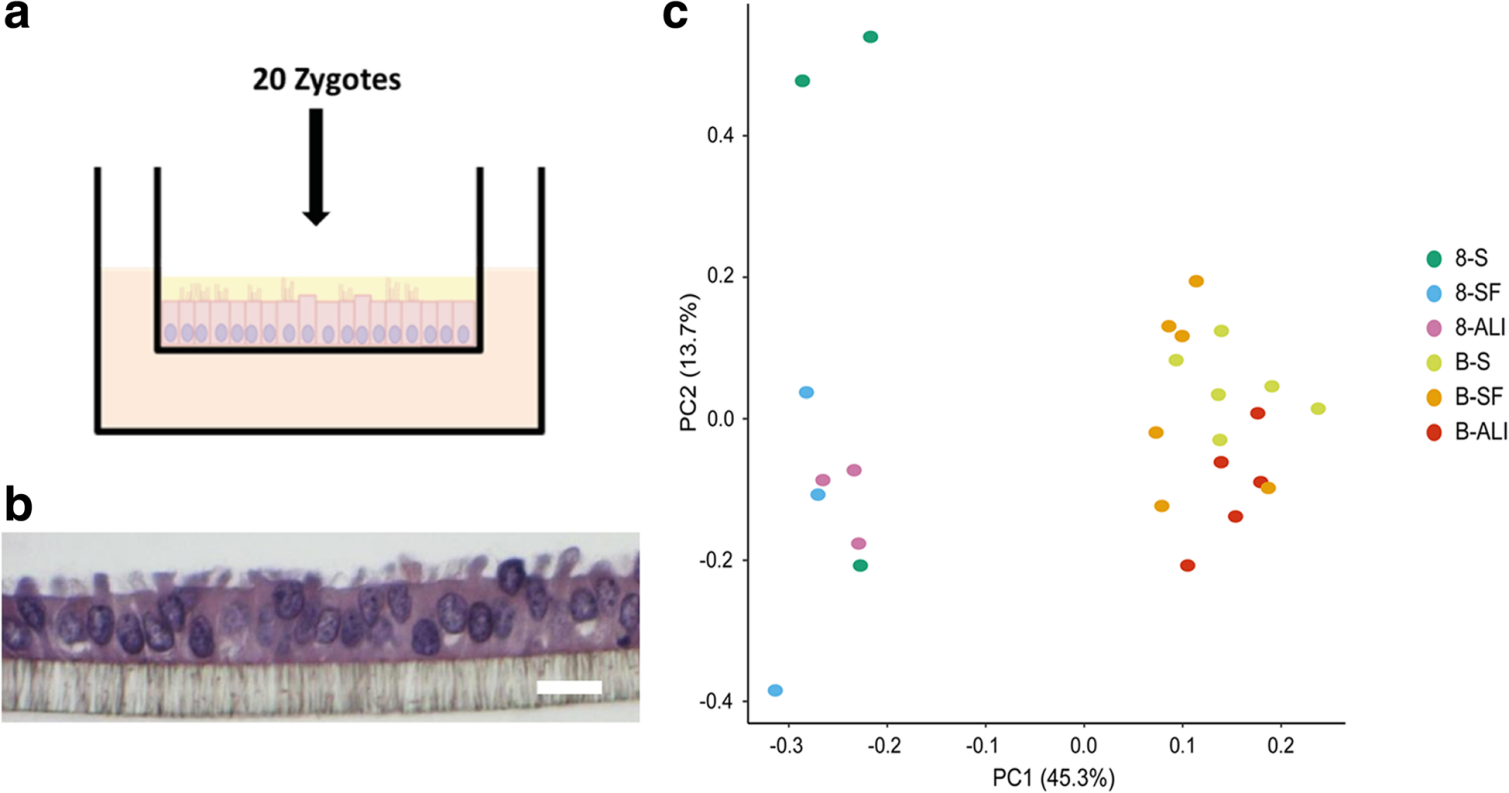
**a** Schematic illustration of the ALI-BOEC co-culture system and **b** representative HE staining of ALI-BOEC after 4 weeks of culture. Magnification 400 x. Bar = 10 μm. **c** Principal Component Analysis plot of gene expression data obtained from bovine preimplantation embryos produced in different in vitro conditions. Principle component 1 explains 45.3% of the variance and principle component 2 explains 13.7% of the variance

1. Standard IVP
  The IVP procedure was performed as described previously ([5]; experiment 1, protocol 2). In trial 1 (collection of 8-cell embryos; *n* = 115 oocytes) cleavage rate was 77.39% (*n* = 89). In trial 2 (collection of blastocysts; *n* = 95 oocytes) cleavage rate was 72.63% (*n* = 69) and blastocyst rate 47.83% (*n* = 33).
2. Commercial serum-free IVP suite
  The IVP procedure was performed using the IVF Bioscience media suite for bovine embryo production according to the manufacturer’s instructions (IVF Bioscience, Falmouth, UK, cat. # 61008, 61002, 61004, 61003, and 61001).
  In trial 1 (*n* = 34 oocytes), the cleavage rate was 97.06% (*n* = 33). In trial 2 (*n* = 73 oocytes), the cleavage rate was 91.78% (*n* = 67) and the blastocyst rate 49.25% (*n* = 33).
3. ALI-BOEC co-culture.
  The protocol for culturing ALI-BOEC on hanging inserts has recently been reported [4] and histological samples to verify differentiation of the cell culture were prepared following published protocols [6]. A scheme of the culture system and a representative histological picture of ALI-BOEC are shown in Fig. 1a and b. The maturation and fertilization of oocytes followed the procedure described in paragraph “1) Standard IVP”. 100 oocytes were applied for trial 1 [cleavage rate 60% (*n* = 60)]. Another 100 oocytes was applied in trial 2 [cleavage rate 72% (*n* = 72) and blastocyst rate 9.72% (*n* = 7)].

### Selection of target genes

Forty-one target genes were selected based on previously published transcriptomics and qRT-PCR data of developing bovine embryos. The PubMed database was used to search papers with the following keywords: ‘bovine embryo development’, ‘transcriptomics’, ‘gene expression’, ‘in vivo’ and ‘in vitro fertilization’. The selection criteria included the availability of gene expression data of different days of embryo development, or differences between various in vitro culture conditions or in vivo. Twenty-six publications (Additional file 1: Table S1) were used for a final selection of 41 target genes. The selected genes were subdivided into five functional categories, i.e. ‘imprinted genes and DNA methylation’, ‘stem cell markers and differentiation’, ‘apoptosis’, ‘embryo development, quality and competence’, and ‘metabolism’.

### cDNA synthesis and specific target amplification

The CellsDirect™ One-Step qRT-PCR Kit (ThermoFisher Scientific, Waltham, MA, USA, cat. # 11753100) was used for cDNA synthesis and specific-target amplification (STA), as described previously [7] with minor modifications. In brief, an STA master mix was prepared, consisting of 5 μl 2× Reaction Mix, 0.2 μl CellsDirect Enzyme Mix, 2.5 μl primer mix, 0.2 μl SUPERase• In™ RNase Inhibitor (20 U/μl) (ThermoFisher Scientific, Waltham, MA, USA, cat. # AM2694), and 1 × TE buffer (ThermoFisher Scientific, Waltham, MA, USA, cat. # 12090015). All primers were ordered from Microsynth (Balgach, Switzerland), and product specificity was assessed based on product size by gel electrophoresis. Gene descriptions and primer sequences are available in the Additional file 2: Table S2. Nine microliter of the STA master mix was added to the frozen embryos, which were then homogenized by means of pipetting. Both reverse transcription and STA were performed in a thermal cycler by incubation for 15 min at 50 °C, followed by 2 min incubation at 95 °C. The STA was performed by 18 cycles of 15 s at 95 °C and 4 min at 60 °C. Reactions were cleaned from residual primers by a treatment with an Exonuclease I master mix, consisting of 0.8 μl Exonuclease I (20 U/μl) (ThermoFisher Scientific, Waltham, MA, USA, cat. # EN0581), 0.4 μl 10× Exonuclease I Reaction Buffer, and 2.8 μl nuclease free water. Four microliter of Exonuclease master mix was added to each STA product and incubated at 37 °C for 15 min followed by heat-inactivation of the enzyme at 80 °C for 15 min.

### Biomark delta gene assay

The STA samples were used for gene expression analysis using a Biomark HD instrument. The Sample Pre-Mix consisted of 3 μl 2× TaqMan Gene Expression Master Mix (Applied Biosystems, Foster City, CA, USA, cat. # PN 4369016), 0.3 μl 20× DNA Binding Dye Sample Loading Reagent (Fluidigm, San Francisco, CA, USA, cat. # PN 100–0388), 0.3 μl 20 × EvaGreen DNA binding dye (Biotium, Fremont, CA, USA, cat. # PN 31000), and 0.9 μl TE buffer. The Sample Pre-Mix was combined with 1.5 μl of 10× diluted cleaned-up STA cDNA. The Assay Mix was prepared for 48 primer pairs, consisting of 3 μl 2× Assay Loading Reagent (Fluidigm, San Francisco, CA, USA, cat. # PN 85000736), 0.3 μl TE buffer, and 2.7 μl of 20 μM of Forward and Reverse Primer Mix. One 48.48 Dynamic Array™ (Fluidigm, San Francisco, CA, USA, cat. # BMK-M-48.48) chip was loaded and run according to manufacturer instructions as described in the Fluidigm Advanced Development Protocol 14. After a hot-start of 120 s at 50 °C and 600 s at 95 °C, the amplification was performed by 40 cycles of 15 s at 95 °C and 60 s at 60 °C. A melting curve was generated by a temperature increase from 60 °C to 95 °C with increments of 1 °C/s.

### Data analysis

The Fluidigm Real-Time PCR Analysis Software was used for quality control of the experiment and for validation of product specificity. A cut-off of Ct = 25 was set as limit of detection. To allow analysis of expression patterns of very low abundant genes a Ct value of 25 was assigned if gene expression levels were lower than the limit of detection. Genes with low expression levels in some samples were *PTGS2, FADS1*, and *HSPA1A* (Additional file 3: Table S4; specific samples with expression levels lower than the limit of detection highlighted in blue). Using the geNorm algorithm within the GenEx6 software, the geometric mean of all reference genes, i.e. UBB, H3F3A, YWHAZ, GAPDH, and SDHA, was found to be the most stable reference [8]. The normalized expression (∆C_t_) values were log2 transformed and centred for both PCA plot and heatmap, which were processed in R Studio. In addition, the differential gene expression from 8-cell embryos to blastocysts was displayed in a heatmap using the log2 transformed fold changes (∆∆Ct).The statistical analysis of differentially expressed genes (DEGs) was performed on ∆C_t_ values in IBM SPSS Statistics 23 using one-way ANOVA with Tukey post-hoc test. Graphs of log_2_ fold-changes were made in GraphPad Prism 7.02.

## Results and discussion

The differentiation status of ALI-BOEC was verified by their polarized morphology and presence of ciliated and secretory sub-populations (Fig. 1b). The overall cleavage rate in ALI-BOEC co-culture (ALI, 66%) was comparable to the standard IVP procedure (S, 75.01%), and lower than the cleavage rate reached with the commercial serum-free media suit (SF, 94.42%). However, the blastocyst rate in ALI-BOEC co-culture (9.72%) was considerably lower than in either IVP-S (47.83%) or the commercial IVP-SF system (49.25%). This may be caused by the fact that in vivo bovine zygotes stay in the oviduct for 3–5 days and only develop up to the 8–16 cell stage before entering the uterus [9]. As the oviductal secretions are greatly influenced by hormones during the oestrous cycle [10], the lack of hormonal stimulation during co-culture may also affect the competence of ALI-BOEC to adequately support embryo development.

**Fig. 2:**
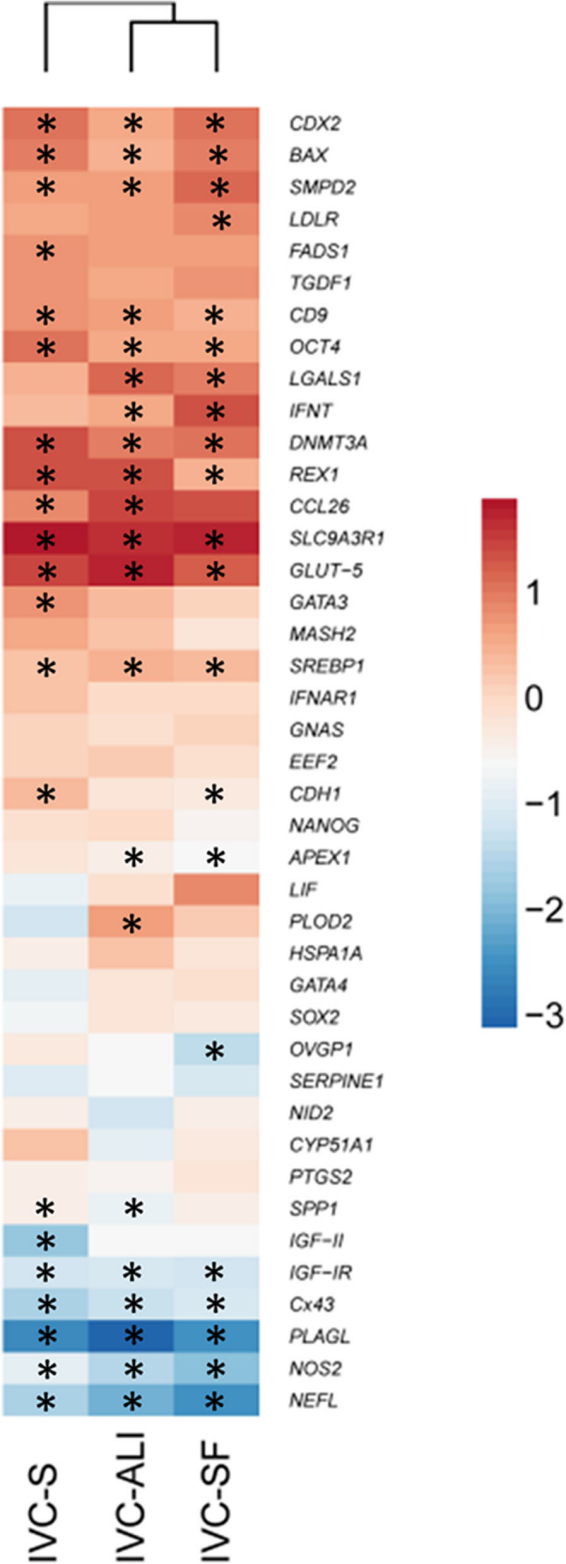
Hierarchical clustering of mean log2 fold-changes of differentially expressed genes between blastocysts and 8-cell embryos. Significant differences are marked * (*p* < 0.05) accordingly

Comparing the viable embryos, the gene expression of embryos produced in the different in vitro systems showed (as expected) a clear separation between 8-cell embryos and blastocysts [11]. However, no culture condition-dependent clustering was observed based on the expression values of all investigated genes, as visualized by principle component analysis (PCA) (Fig. 1c) and hierarchical clustering (Additional file 4: Figure S1). Furthermore, the embryos from all three culture conditions were largely similar in their differential target gene expression from the 8-cell to the blastocyst stage (Fig. 2).

**Fig. 3:**
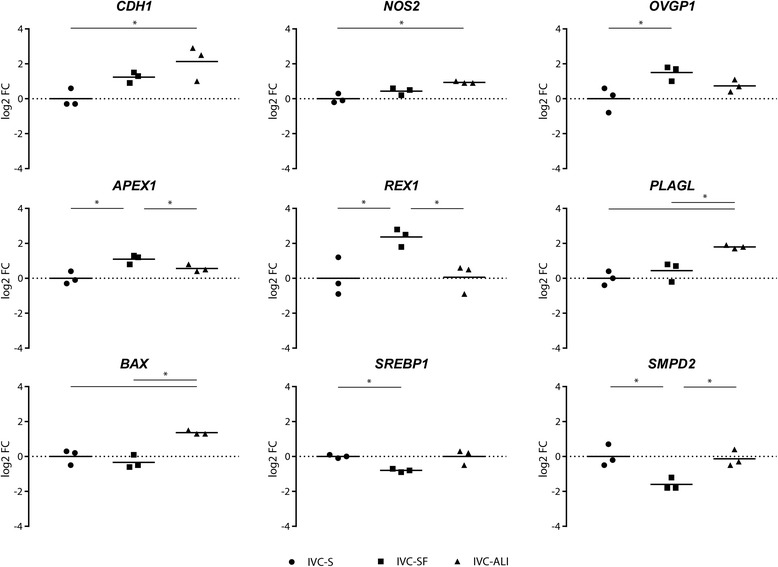
Differentially expressed genes in 8-cell stage embryos. The log2 fold-change of the mRNA expression is displayed for each treatment group (*n* = 3 pools of 5 embryos). Different groups are compared by one-way ANOVA. Significant differences are marked * (*p* < 0.05) accordingly

**Fig. 4:**
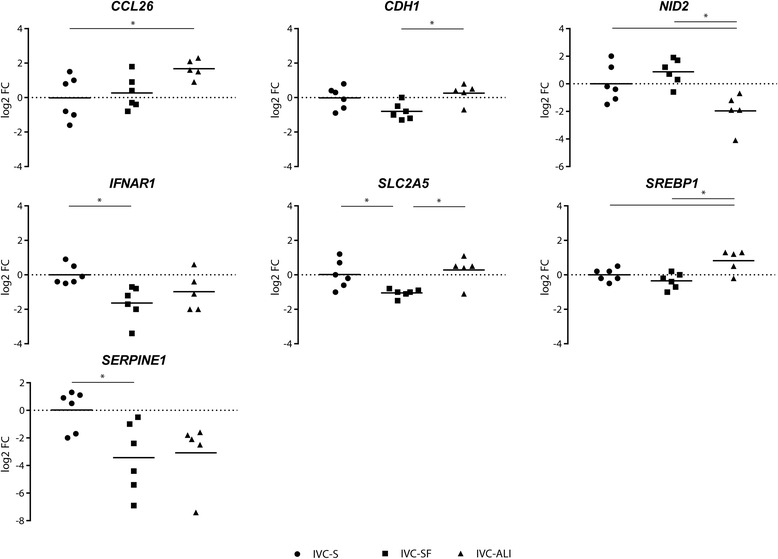
Differentially expressed genes in blastocysts. The log2 fold-change of the mRNA expression is displayed for each treatment group (*n* = 6 single blastocysts for IVC Standard and IVC Bioscience, and *n* = 5 single blastocysts for ALI-BOEC). Different groups are compared by one-way ANOVA. Significant differences are marked * (*p* < 0.05) accordingly

Among the culture conditions, nine and seven genes showed a statistically significant differential expression for the 8-cell embryos (Fig. 3), and the blastocysts (Fig. 4), respectively. In the 8-cell embryos most of the DEGs, namely *CDH1*, *NOS2*, *APEX1*, *REX1*, *PLAGL1*, and *BAX*, have been associated with embryo quality and competence or apoptosis regulation [12–17]. ALI-BOEC embryos were divergent from both other groups in their expression of *PLAGL* and *BAX*, two major regulators of apoptosis. We conclude that 8-cell embryos cultured on ALI-BOEC might be less developmentally active and have higher levels of embryonic growth control and apoptosis, which is in line with the relatively low blastocyst rate in this group. In contrast, *APEX1*, a gene involved in DNA repair [18] and *REX1*, which is regulating stem cell pluripotency [19] were significantly higher expressed in IVC-SF embryos than in embryos from the other two groups. Together with the lower expression of *SMPD2* in IVC-SF embryos, which is connected to growth arrest and apoptosis [20], this expression pattern is indicative for an increased developmental activity and therefore in line with the high cleavage and blastocyst rate in the embryos developing in the commercial serum-free media suit (IVC-SF).

Surprisingly, the mRNA expression of *OVGP1*, a marker gene for OEC [21], was detected in all 8-cell embryo groups. Initially chosen as a target gene to notice any contamination of the ALI-BOEC embryos with epithelial cells, the expression of *OVGP1* mRNA was significantly higher in embryos cultured in commercial serum-free media compared to ALI and was also detectable in standard IVP embryos. The results indicate that a) *OVGP1* mRNA is actively expressed in IVP embryos and b) OEC do not necessarily contribute to this expression. The presence and significance of *OVGP1* expression in IVP embryos requires further analysis.

The differentially expressed genes in *blastocysts* derived from the different IVP systems either have a function in embryo development, quality, and competence (*CCL26*, *CDH1*, *NID2*, and *IFNAR1*) [22–26] or in embryonic metabolism (*SLC2A5*, *SREBP1*, *SERPINE1*) [27–29]. Eighteen of our target genes were previously reported to be differentially expressed between in vivo and in vitro blastocysts (Additional file 5: Table S3) and could therefore be indicative for more in vivo-like embryonic development. However, only three of these genes are among the differentially expressed genes in our analysis (CDH1, NID2 and SLC2A5). ALI-OEC blastocysts show significant lower NID2 expression compared to both conventional IVP groups. In vivo embryos are reported to have lower expression of NID2 compared to embryos produced in conventional IVP systems as well (Additional file 5: Table S3). Likewise, IVP-SF embryos, displayed differential expression of SLC2A5, a fructose transporter, compared to both other groups. SLC2A5 has previously been reported to be higher expressed in vivo than in in vitro embryos. IVP-SF embryos, however, showed a lower expression of this gene. CDH1 expression was significantly different only between ALI and IVC-S embryos.

## Conclusion

The ALI-BOEC co-culture system was much less efficient in supporting blastocyst formation than optimized conventional IVP procedures. The different culture conditions lead to differential gene expression in both 8-cell embryos and blastocysts. However, across the expression of all target genes, the embryos developing on ALI-BOEC did not clearly depart from conventional IVP embryos. Our results neither hint for largely aberrant, nor for more in vivo-like gene expression of embryos produced in co-culture with ALI-BOEC. To further optimize the ALI-BOEC system we propose to develop a dynamic hormonal (progesterone) stimulation protocol mimicking the hormonal environment in vivo. Furthermore, the establishment of a sequential culture system of oviductal and uterine epithelial cells might increase the efficiency of the production system not only quantitatively, but also qualitatively in view of its competence to support more in vivo-like embryonic development.

## Additional files

Additional file 1: Table S1. References for target gene selection. (DOC 74 kb)

Additional file 2: Table S2. Target gene descriptions and primer sequences. (DOC 154 kb)

Additional file 3: Table S4. Full data set of expression profiles from in vitro derived embryos. (XLS 68 kb)

Additional file 4: Figure S1. Hierarchical clustering of gene expression data obtained from bovine preimplantation embryos produced in different in vitro conditions. (TIFF 2547 kb)

Additional file 5: Table S3. Previously reported differential expression of target genes between in vivo and in vitro produced blastocysts in comparison to expression patterns in ALI derived embryos. (DOC 54 kb)
