# Supplementary material for: Gene expression of bovine embryos developing at the air-liquid interface on oviductal epithelial cells (ALI-BOEC)
Source: Reprod Biol Endocrinol. 2017 Nov 25;15:91. doi: 10.1186/s12958-017-0310-1 (PMC5702211; doi:10.1186/s12958-017-0310-1)
Supplement: Supplementary file 1 — References for target gene selection. (DOC 74 kb) [file 12958_2017_310_MOESM1_ESM.doc]

**Additional file 1: Table S1.** References for target gene selection

| **Gene** | **Gene description** | **NCBI Reference Sequence** | **Reference** |
| --- | --- | --- | --- |
| IFNT | Interferon tau | NM_001015511.3 | [1-3] |
| BAX | BCL2-associated X protein | NM_173894.1 | [1, 4] |
| TDGF1 | Teratocarcinoma-derived growth factor 1 | NM_001080358.1 | [5, 6] |
| SLC9A3R1 | Solute carrier family 9, subfamily A (NHE3, cation proton antiporter 3), member 3 regulator 1 | NM_001077852.2 | [6, 7] |
| Cx43/GJA | Connexin43 | NM_174068.2 | [5, 8, 9] |
| PLAGL1 | Pleiomorphic adenoma gene-like 1 | NM_001103289.1 | [7, 10] |
| GNAS | GNAS complex locus | NM_001271771.1 | [7, 10] |
| DNMT3A | DNA (cytosine-5-)-methyltransferase 3 alpha | NM_001206502.1 | [7, 10] |
| GATA3 | GATA binding protein 3 | NM_001076804.1 | [6, 11, 12] |
| CCL26 | Chemokine (C-C motif) ligand 26 | NM_001205635.1 | [6, 7, 12] |
| CYP51A1 | Cytochrome P450  family 51, subfamily A, polypeptide 1 | NM_001025319.2 | [5] |
| FADS1 | Fatty acid desaturase 1 | XM_005226961.3 | [5, 7] |
| CD9 | Member of tetraspanin family | NM_173900.2 | [6, 13] |
| LGALS1 | Lectin, Galactoside Binding Soluble 1 | NM_175782.1 | [6] |
| EEF2 | Eukaryotic Translation Elongation Factor 2 | NM_001075121.1 | [6] |
| PTGS2 | Prostaglandin-Endoperoxide Synthase 2 | NM_174445.2 | [6] |
| GLUT-5 | Fructose transporter | NM_001101042.2 | [1] |
| IGF-II | Insulin-like growth factor | NM_174087.3 | [1] |
| SOX2 | SRY-related HMG-box | NM_001105463.2 | [14] |
| IGF-IR | Insulin-like growth factor 1 (IGF-1) receptor | NM_001244612.1 | [1] |
| SPP1 | Secreted phosphoprotein 1 | NM_174187.2 | [15, 16] |
| NEFL | Neurofilament, light polypeptide | NM_174121.1 | [5, 17] |
| NID2 | Nidogen 2 (osteonidogen) | NM_001102065.1 | [5, 18] |
| NOS2 | Nitric oxide synthase 2, inducible | NM_001076799.1 | [5, 19] |
| PLOD2 | Procollagen-lysine, 2-oxoglutarate 5-dioxygenase 2 | NM_001101149.1 | [5, 20] |
| LDLR | Low density lipoprotein receptor | NM_001166530.1 | [5, 21] |
| IFNAR1 | Interferon Alpha And Beta Receptor Subunit 1 | NM_174552.2 | [2, 8, 22] |
| MASH2 | Mammalian achaetescute homologue | NM_001040607.1 | [18, 23] |
| HSPA1A | Heat shock protein 70.1 | NM_174550.1 | [23] |
| SERPINE1 | Serpin family E member 1 | NM_174137.2 | [21, 24] |
| SREBP1 | Sterol regulatory element-binding protein 1 | NM_001113302.1 | [21, 25] |
| APEX1 | Apurinic/apyrimidinic endodeoxyribonuclease 1 | NM_176609.3 | [21] |
| SMPD2 | Sphingomyelin phosphodiesterase 2 | NM_001075383.2 | [24] |
| LIF | Leukemia inhibitory factor | XM_015475541.1 | [4] |
| CDH1 | E-cadherin | NM_001002763.1 | [23, 26] |
| OCT4 | Octamer-binding transcription factor 4 | NM_174580.3 | [14] |
| CDX2 | Caudal type homeobox 2 | NM_001206299.1 | [14] |
| REX1 | ZFP42  zinc finger protein 42 | XM_003587951.3 | [14] |
| GATA4 | GATA binding protein 4 | NM_001192877.1 | [14] |
| NANOG | Nanog homeobox | NM_001025344.1 | [14] |
| OVGP1 | Oviductal glycoprotein 1 | XM_005204074.3 | Marker gene for OEC |
| UBB | Ubiquitin B | NM_174133.2 | Reference gene |
| H3F3A | H3 Histone Family Member 3A | NM_001014389.2 | Reference gene |
| YWHAZ | Tyrosine 3-Monooxygenase/Tryptophan 5-Monooxygenase Activation Protein Zeta | NM_174814.2 | Reference gene [27] |
| SDHA | Succinate dehydrogenase complex flavoprotein subunit A | NM_174178 | Reference gene [27] |
| GAPDH | Glyceraldehyde 3-phosphate dehydrogenase | NM_001034034.2 | Reference gene [27] |

Reference List

1. Lonergan P, Rizos D, Gutierrez-Adan A, Moreira PM, Pintado B, de la Fuente J, Boland MP. Temporal divergence in the pattern of messenger RNA expression in bovine embryos cultured from the zygote to blastocyst stage in vitro or in vivo. *Biol Reprod* 2003, 69:1424-1431.

2. Takahashi M, Takahashi H, Hamano S, Watanabe S, Inumaru S, Geshi M, Okuda K, Yokomizo Y, Okano A. Possible role of interferon-tau on in vitro development of bovine embryos. *J Reprod Dev* 2003, 49:297-305.

3. Wrenzycki C, Herrmann D, Keskintepe L, Martins A, Jr., Sirisathien S, Brackett B, Niemann H. Effects of culture system and protein supplementation on mRNA expression in pre-implantation bovine embryos. *Hum Reprod* 2001, 16:893-901.

4. Rizos D, Lonergan P, Boland MP, Arroyo-Garcia R, Pintado B, de la Fuente J, Gutierrez-Adan A. Analysis of differential messenger RNA expression between bovine blastocysts produced in different culture systems: implications for blastocyst quality. *Biol Reprod* 2002, 66:589-595.

5. Clemente M, Lopez-Vidriero I, O'Gaora P, Mehta JP, Forde N, Gutierrez-Adan A, Lonergan P, Rizos D. Transcriptome changes at the initiation of elongation in the bovine conceptus. *Biol Reprod* 2011, 85:285-295.

6. Jiang Z, Sun J, Dong H, Luo O, Zheng X, Obergfell C, Tang Y, Bi J, O'Neill R, Ruan Y, et al. Transcriptional profiles of bovine in vivo pre-implantation development. *BMC Genomics* 2014, 15:756.

7. Graf A, Krebs S, Zakhartchenko V, Schwalb B, Blum H, Wolf E. Fine mapping of genome activation in bovine embryos by RNA sequencing. *Proc Natl Acad Sci U S A* 2014, 111:4139-4144.

8. Bao ZJ, Zhao S, Haq IU, Zeng SM. Recombinant bovine interferon-tau enhances in vitro development of bovine embryos by upregulating expression of connexin 43 and E-cadherin. *J Dairy Sci* 2014, 97:6917-6925.

9. De Sousa PA, Juneja SC, Caveney S, Houghton FD, Davies TC, Reaume AG, Rossant J, Kidder GM. Normal development of preimplantation mouse embryos deficient in gap junctional coupling. *J Cell Sci* 1997, 110 ( Pt 15):1751-1758.

10. Jiang Z, Dong H, Zheng X, Marjani SL, Donovan DM, Chen J, Tian XC. mRNA Levels of Imprinted Genes in Bovine In Vivo Oocytes, Embryos and Cross Species Comparisons with Humans, Mice and Pigs. *Sci Rep* 2015, 5:17898.

11. Bai H, Sakurai T, Someya Y, Konno T, Ideta A, Aoyagi Y, Imakawa K. Regulation of trophoblast-specific factors by GATA2 and GATA3 in bovine trophoblast CT-1 cells. *J Reprod Dev* 2011, 57:518-525.

12. Smith SL, Everts RE, Tian XC, Du F, Sung LY, Rodriguez-Zas SL, Jeong BS, Renard JP, Lewin HA, Yang X. Global gene expression profiles reveal significant nuclear reprogramming by the blastocyst stage after cloning. *Proc Natl Acad Sci U S A* 2005, 102:17582-17587.

13. Xiang W, MacLaren LA. Expression of fertilin and CD9 in bovine trophoblast and endometrium during implantation. *Biol Reprod* 2002, 66:1790-1796.

14. Hajian M, Hosseini SM, Ostadhosseini S, Nasr-Esfahani MH. Targeting the transforming growth factor-beta signaling during pre-implantation development in embryos of cattle, sheep and goats. *Growth Factors* 2016, 34:141-148.

15. Kues WA, Sudheer S, Herrmann D, Carnwath JW, Havlicek V, Besenfelder U, Lehrach H, Adjaye J, Niemann H. Genome-wide expression profiling reveals distinct clusters of transcriptional regulation during bovine preimplantation development in vivo. *Proc Natl Acad Sci U S A* 2008, 105:19768-19773.

16. Wang X, Johnson GA, Burghardt RC, Wu G, Bazer FW. Uterine Histotroph and Conceptus Development. II. Arginine and Secreted Phosphoprotein 1 Cooperatively Stimulate Migration and Adhesion of Ovine Trophectoderm Cells via Focal Adhesion-MTORC2 Mediated Cytoskeleton Reorganization. *Biol Reprod* 2016, 95:71.

17. Kim EY, Lee KB, Yu J, Lee JH, Kim KJ, Han KW, Park KS, Lee DS, Kim MK. Neuronal cell differentiation of mesenchymal stem cells originating from canine amniotic fluid. *Hum Cell* 2014, 27:51-58.

18. Marchand M, Horcajadas JA, Esteban FJ, McElroy SL, Fisher SJ, Giudice LC. Transcriptomic signature of trophoblast differentiation in a human embryonic stem cell model. *Biol Reprod* 2011, 84:1258-1271.

19. Kobayashi Y, Yamamoto Y, Kageyama S, Hirayama H, Kimura K, Okuda K. Regulation of bovine oviductal NO synthesis by follicular steroids and prostaglandins. *Reproduction* 2016, 151:577-587.

20. Venables JP, Lapasset L, Gadea G, Fort P, Klinck R, Irimia M, Vignal E, Thibault P, Prinos P, Chabot B, et al. MBNL1 and RBFOX2 cooperate to establish a splicing programme involved in pluripotent stem cell differentiation. *Nat Commun* 2013, 4:2480.

21. Cagnone G, Sirard MA. The impact of exposure to serum lipids during in vitro culture on the transcriptome of bovine blastocysts. *Theriogenology* 2014, 81:712-722 e711-713.

22. Payelle-Brogard B, Pellegrini S: Biochemical monitoring of the early endocytic traffic of the type I interferon receptor. *J Interferon Cytokine Res* 2010, 30:89-98.

23. Wrenzycki C, Wells D, Herrmann D, Miller A, Oliver J, Tervit R, Niemann H. Nuclear transfer protocol affects messenger RNA expression patterns in cloned bovine blastocysts. *Biol Reprod* 2001, 65:309-317.

24. de Castro e Paula LA, Hansen PJ. Ceramide inhibits development and cytokinesis and induces apoptosis in preimplantation bovine embryos. *Mol Reprod Dev* 2008, 75:1063-1070.

25. Vergnes L, Chin RG, de Aguiar Vallim T, Fong LG, Osborne TF, Young SG, Reue K. SREBP-2-deficient and hypomorphic mice reveal roles for SREBP-2 in embryonic development and SREBP-1c expression. *J Lipid Res* 2016, 57:410-421.

26. Tesfaye D, Lonergan P, Hoelker M, Rings F, Nganvongpanit K, Havlicek V, Besenfelder U, Jennen D, Tholen E, Schellander K. Suppression of connexin 43 and E-cadherin transcripts in in vitro derived bovine embryos following culture in vitro or in vivo in the homologous bovine oviduct. *Mol Reprod Dev* 2007, 74:978-988.

27. Goossens K, Van Poucke M, Van Soom A, Vandesompele J, Van Zeveren A, Peelman LJ. Selection of reference genes for quantitative real-time PCR in bovine preimplantation embryos. *BMC Dev Biol* 2005, 5:27.
