# Supplementary material for: Gene expression of bovine embryos developing at the air-liquid interface on oviductal epithelial cells (ALI-BOEC)
Source: Reprod Biol Endocrinol. 2017 Nov 25;15:91. doi: 10.1186/s12958-017-0310-1 (PMC5702211; doi:10.1186/s12958-017-0310-1)
Supplement: Supplementary file 2 — Target gene descriptions and primer sequences. (DOC 154 kb) [file 12958_2017_310_MOESM2_ESM.doc]

**Additional file 2: Table S2.** Primer sequences

| **Gene** | **Gene description** | **NCBI Reference Sequence** | **Direction** | **Sequence (5’ 🡪 3’)** | **Ref.** |
| --- | --- | --- | --- | --- | --- |
| IFNT | interferon tau | NM_001015511.3 | Forward | GGATAATGAGTACCGTCTTCCC |  |
|  |  |  | Reverse | TGAGATGGGGTCTGGGTGAA |  |
| BAX | BCL2-associated X protein | NM_173894.1 | Forward | GCCCTTTTGCTTCAGGGTTT |  |
|  |  |  | Reverse | ACAGCTGCGATCATCCTCTG |  |
| TDGF1 | teratocarcinoma-derived growth factor 1 | NM_001080358.1 | Forward | TCATGCAGATTTCATGACCTG | [1] |
|  |  |  | Reverse | TTTAGGGACCACAGGGAAATC |  |
| SLC9A3R1 | solute carrier family 9, subfamily A (NHE3, cation proton antiporter 3), member 3 regulator 1 | NM_001077852.2 | Forward | TGTTTGATCCCCGGCTTCG |  |
|  |  |  | Reverse | CCAACAAGCGAGCAGGTGT |  |
| Cx43/GJA | Connexin43 | NM_174068.2 | Forward | GTTGCCCAAACTGATGGTG | [2] |
|  |  |  | Reverse | TAGGTTCTCAGCAAGCCCC |  |
| PLAGL1 | pleiomorphic adenoma gene-like 1 | NM_001103289.1 | Forward | TTCCCTGCCTACTTGAGGGA |  |
|  |  |  | Reverse | TAAGCTTGGGGTCCAGAGGA |  |
| GNAS | GNAS complex locus | NM_001271771.1 | Forward | CCCCTTTTCCCATCCCTTCTT |  |
|  |  |  | Reverse | CTAGAGCCGAAAAAGGTGCC |  |
| DNMT3A | DNA (cytosine-5-)-methyltransferase 3 alpha | NM_001206502.1 | Forward | CAAAGTGAGGACCATTACTACGA | [3] |
|  |  |  | Reverse | CCGAACACCCTTTCCATCTC |  |
| GATA3 | GATA binding protein 3 | NM_001076804.1 | Forward | CGGGAGTGTGTGAACTGTGG |  |
|  |  |  | Reverse | TGCAGACAGGGTCTCCATTG |  |
| CCL26 | chemokine (C-C motif) ligand 26 | NM_001205635.1 | Forward | TTGAGTGTCGATCTCGGAGC |  |
|  |  |  | Reverse | GCACCCATTCTTCCTTTGGC |  |
| CYP51A1 | cytochrome P450  family 51, subfamily A, polypeptide 1 | NM_001025319.2 | Forward | GGGGCGAAAAGTCCACCATA |  |
|  |  |  | Reverse | CCCCAGCAGGTAGGTAAACG |  |
| FADS1 | fatty acid desaturase 1 | XM_005226961.3 | Forward | GAATTCCAGCAGGAGCGAGA |  |
|  |  |  | Reverse | GTTCATCCGTAGCCCGAGAC |  |
| CD9 | member of tetraspanin family | NM_173900.2 | Forward | CCAACCCAGACCACTCTAGC |  |
|  |  |  | Reverse | AGATGCTCTTGGTCTGCGAG |  |
| LGALS1 | Lectin, Galactoside Binding Soluble 1 | NM_175782.1 | Forward | AACAACCTGTGCCTCCACTT |  |
|  |  |  | Reverse | TGGTTAGGTCCGTCTGGTTG |  |
| EEF2 | Eukaryotic Translation Elongation Factor 2 | NM_001075121.1 | Forward | GGCAAATCCACTCTGACGGA |  |
|  |  |  | Reverse | GGAGATGGCCGTTGACTTGA |  |
| PTGS2 | Prostaglandin-Endoperoxide Synthase 2 | NM_174445.2 | Forward | ATCGCAAACGTTTTCTCGTG |  |
|  |  |  | Reverse | CCGAAAGTGCTAGGCTTCCA |  |
| GLUT-5 | Fructose transporter | NM_001101042.2 | Forward | TTCCAATGTCGTCCCCATGT |  |
|  |  |  | Reverse | CAGTCAATCCGAGGAGGATGG |  |
|  |  |  | Reverse | GAGGTTGTTCACGTAGGCCG |  |
| IGF-II | Insulin-like growth factor | NM_174087.3 | Forward | GTGCTTCTTGCCTTCTTGGC |  |
|  |  |  | Reverse | TGGTCGGCTGAAGTAGAAGC |  |
| SOX2 | SRY-related HMG-box | NM_001105463.2 | Forward | CGCCCTGCAGTACAACTCTA |  |
|  |  |  | Reverse | GGGTGCCCTGCTGAGAATAG |  |
| IGF-IR | Insulin-like growth factor 1 (IGF-1) receptor | NM_001244612.1 | Forward | GATCCCGTGTTCTTCTACGTTC |  |
|  |  |  | Reverse | AAGCCTCCCACTATCAACAGAA |  |
| SPP1 | Secreted phosphoprotein 1 | NM_174187.2 | Forward | GCCACAGAGGAGGACTTCAC |  |
|  |  |  | Reverse | TTATCCTTGGCCTTTGGCGT |  |
| NEFL | neurofilament, light polypeptide | NM_174121.1 | Forward | GCAGATCCAGTACGCACAGA |  |
|  |  |  | Reverse | CAGGGTCTTGGCCTTAAGCA |  |
| NID2 | nidogen 2 (osteonidogen) | NM_001102065.1 | Forward | GACAGATGTTGCTGGACGGA |  |
|  |  |  | Reverse | GCAAGACCTTCGGGGCTTAT |  |
| NOS2 | nitric oxide synthase 2, inducible | NM_001076799.1 | Forward | ACCATGAGGCTCAAATCGCA |  |
|  |  |  | Reverse | CCTTGACCCAATAGCTGCCA |  |
| PLOD2 | procollagen-lysine, 2-oxoglutarate 5-dioxygenase 2 | NM_001101149.1 | Forward | GTCCTTAAGTCAAGACAGGGTCA |  |
|  |  |  | Reverse | GGGAAGAACAAAGGCTCACCA |  |
| LDLR | low density lipoprotein receptor | NM_001166530.1 | Forward | AATTCCAGTGCTCGGATGGG |  |
|  |  |  | Reverse | GTTGCACACTTTGTCCAGGG |  |
| IFNAR1 | Interferon Alpha And Beta Receptor Subunit 1 | NM_174552.2 | Forward | CTCCAGTCATCAGCGTGAAA |  |
|  |  |  | Reverse | GTGCTCTGGCTTTGACACAA |  |
| MASH2 | mammalian achaetescute homologue | NM_001040607.1 | Forward | CCTGACCCAAGGCTAGTGTG |  |
|  |  |  | Reverse | TTGCGTCGTCATAAAGCCCT |  |
| HSPA1A | heat shock protein 70.1 | NM_174550.1 | Forward | GGGCACCAGAGCTTCACGAT |  |
|  |  |  | Reverse | GTCGATGCCGATAGCCATGT |  |
| SERPINE1 | serpin family E member 1 | NM_174137.2 | Forward | CTGCGAAATTCAGGATGCGG |  |
|  |  |  | Reverse | GGGTGAGAAAACCACGTTGC |  |
| SREBP1 | sterol regulatory element-binding protein 1 | NM_001113302.1 | Forward | CACGGTGCCACTGGTAGTAG |  |
|  |  |  | Reverse | TAGCGTTTCTCGATGGCGTT |  |
| APEX1 | apurinic/apyrimidinic endodeoxyribonuclease 1 | NM_176609.3 | Forward | CCGAAACGTGGGAAAAAGGG |  |
|  |  |  | Reverse | ATTTGCCACTGGGTGAGGTT |  |
| SMPD2 | sphingomyelin phosphodiesterase 2 | NM_001075383.2 | Forward | CCCAGCTGCACACTACTTCA |  |
|  |  |  | Reverse | ACGTAGGCATTGAGCACCAA |  |
| LIF | leukemia inhibitory factor | XM_015475541.1 | Forward | GCTTGTGCAGCAAGTACCAC |  |
|  |  |  | Reverse | CTGCTTGTACTTCCCCAGGA |  |
| CDH1 | E-cadherin | NM_001002763.1 | Forward | ACTGCCCCCAGAAGATGAC | [4] |
|  |  |  | Reverse | TCATTGCGAGTCACTTCAGG |  |
| OCT4 | octamer-binding transcription factor 4 | NM_174580.3 | Forward | GAAAGACGTGGTCCGAGTGT |  |
|  |  |  | Reverse | GACAGACACCGAGGGAAAGG |  |
| CDX2 | caudal type homeobox 2 | NM_001206299.1 | Forward | CTTCCCTGCAAGGCTCAGTG |  |
|  |  |  | Reverse | CACTGGGTGACAGTAGGGTT |  |
| REX1 | ZFP42  zinc finger protein 42 | XM_003587951.3 | Forward | TCCTCACAACGGATGCACAA |  |
|  |  |  | Reverse | TTTCCGCACCCTTGGAAAGT |  |
| GATA4 | GATA binding protein 4 | NM_001192877.1 | Forward | TTGTTGGACGTGTGTTGGGA |  |
|  |  |  | Reverse | TGCCAGGTAGCGAGTTTGAG |  |
| NANOG | Nanog homeobox | NM_001025344.1 | Forward | CAAGCCCCAGAGTGAAACCA |  |
|  |  |  | Reverse | GGGTCTGCGAGAACACAGTT |  |
| OVGP1 | Oviductal glycoprotein 1 | XM_005204074.3 | Forward | CACCCACCTGGTATTTGCCT |  |
|  |  |  | Reverse | GGTTGGAAAACGTGGACAGC |  |
| UBB | Ubiquitin B | NM_174133.2 | Forward | AGATCCAGGATAAGGAAGGCAT | [3] |
|  |  |  | Reverse | GCTCCACCTCCAGGGTGAT |  |
| H3F3A | H3 Histone Family Member 3A | NM_001014389.2 | Forward | ACTGGCTACAAAAGCCGCTC | [3] |
|  |  |  | Reverse | ACTTGCCTCCTGCAAAGCAC |  |
| YWHAZ | Tyrosine 3-Monooxygenase/Tryptophan 5-Monooxygenase Activation Protein Zeta | NM_174814.2 | Forward | AGGCTGAGCGATATGATGAC | [3] |
|  |  |  | Reverse | GACCCTCCAAGATGACCTAC |  |
| SDHA | Succinate dehydrogenase complex flavoprotein subunit A | NM_174178 | Forward | GCAGAACCTGATGCTTTGTG | [5] |
|  |  |  | Reverse | CGTAGGAGAGCGTGTGCTT |  |
| GAPDH | Glyceraldehyde 3-phosphate dehydrogenase | NM_001034034.2 | Forward | GGTCACCAGGGCTGCTTTTA |  |
|  |  |  | Reverse | CCAGCATCACCCCACTTGAT |  |

**References**

1. Mitko K, Ulbrich SE, Wenigerkind H, Sinowatz F, Blum H, Wolf E, Bauersachs S. Dynamic changes in messenger RNA profiles of bovine endometrium during the oestrous cycle. *Reproduction* 2008, 135:225-240.

2. Bauersachs S, Ulbrich SE, Zakhartchenko V, Minten M, Reichenbach M, Reichenbach HD, Blum H, Spencer TE, Wolf E. The endometrium responds differently to cloned versus fertilized embryos. *Proc Natl Acad Sci U S A* 2009, 106:5681-5686.

3. Kradolfer D, Floter VL, Bick JT, Furst RW, Rode K, Brehm R, Henning H, Waberski D, Bauersachs S, Ulbrich SE. Epigenetic effects of prenatal estradiol-17beta exposure on the reproductive system of pigs. *Mol Cell Endocrinol* 2016, 430:125-137.

4. Bauersachs S, Ulbrich SE, Reichenbach HD, Reichenbach M, Buttner M, Meyer HH, Spencer TE, Minten M, Sax G, Winter G, Wolf E. Comparison of the effects of early pregnancy with human interferon, alpha 2 (IFNA2), on gene expression in bovine endometrium. *Biol Reprod* 2012, 86:46.

5. Goossens K, Van Poucke M, Van Soom A, Vandesompele J, Van Zeveren A, Peelman LJ. Selection of reference genes for quantitative real-time PCR in bovine preimplantation embryos. *BMC Dev Biol* 2005, 5:27.
