# Supplementary figures and images for: Gene expression of bovine embryos developing at the air-liquid interface on oviductal epithelial cells (ALI-BOEC)
Source: Reprod Biol Endocrinol. 2017 Nov 25;15:91. doi: 10.1186/s12958-017-0310-1 (PMC5702211; doi:10.1186/s12958-017-0310-1)

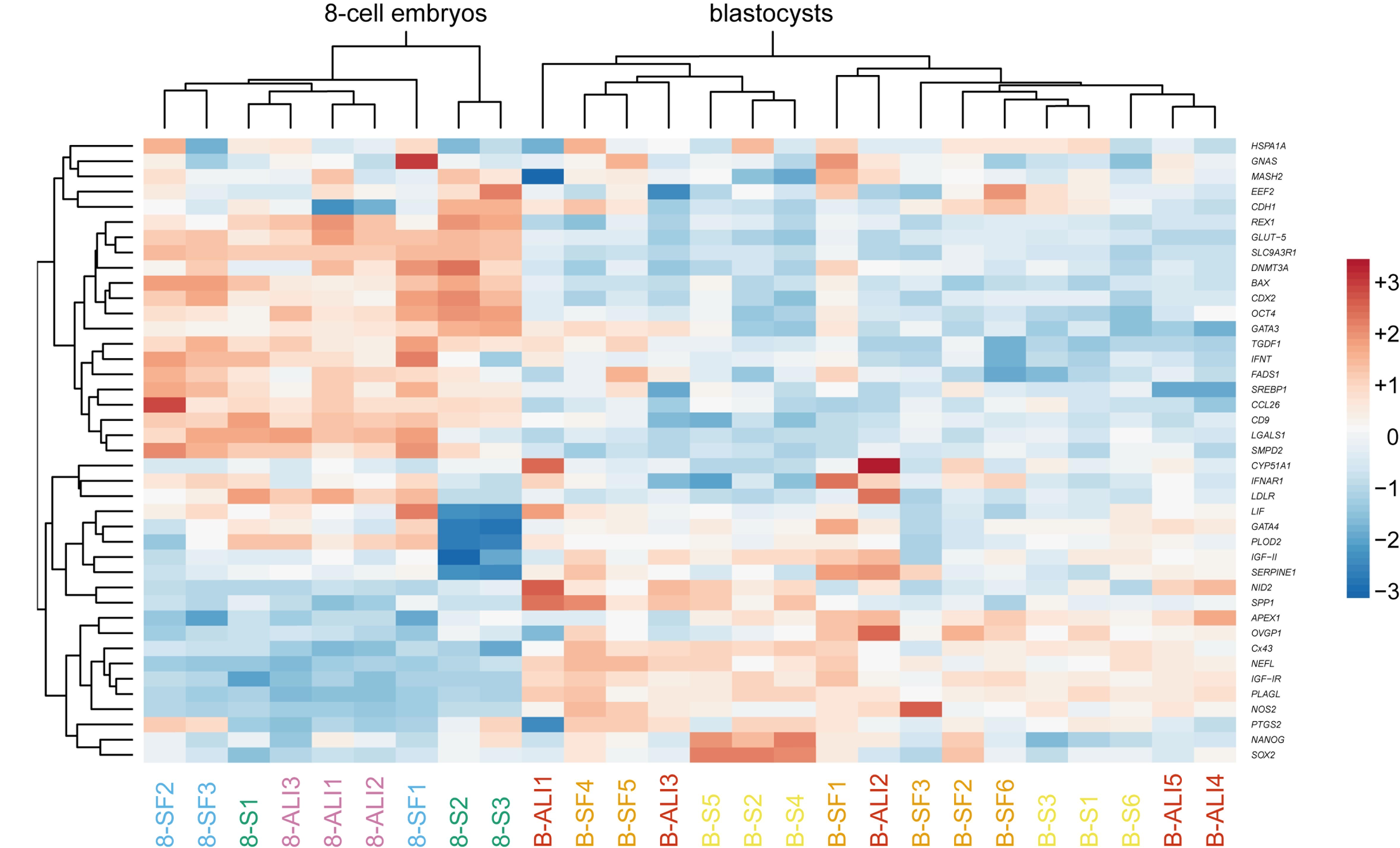

Supplement: Supplementary file 4 — Hierarchical clustering of gene expression data obtained from bovine preimplantation embryos produced in different in vitro conditions. (TIFF 2547 kb) [file 12958_2017_310_MOESM4_ESM.tif]
