# Supplementary material for: Gene expression of bovine embryos developing at the air-liquid interface on oviductal epithelial cells (ALI-BOEC)
Source: Reprod Biol Endocrinol. 2017 Nov 25;15:91. doi: 10.1186/s12958-017-0310-1 (PMC5702211; doi:10.1186/s12958-017-0310-1)
Supplement: Supplementary file 5 — Previously reported differential expression of target genes between in vivo and in vitro produced blastocysts in comparison to expression patterns in ALI derived embryos. (DOC 54 kb) [file 12958_2017_310_MOESM5_ESM.doc]

**Additional file 5: Table S3. Previously reported differential expression of target genes between *in vivo* and *in vitro* produced blastocysts in comparison to expression patterns in ALI derived embryos.**

| **Gene** | **Gene description** | **Expression *in vivo* vs *in vitro* blastocyst** | **Reference** | **ALI-BOEC co-culture vs. conventional *in vitro* systems** |
| --- | --- | --- | --- | --- |
| PLOD2 | procollagen-lysine, 2-oxoglutarate 5-dioxygenase 2 | lower | [1] | ns |
| GATA3 | GATA binding protein 3 | higher | [2] | ns |
| LDLR | low density lipoprotein receptor | lower | [1] | ns |
| TGDF1 | teratocarcinoma-derived growth factor 1 | higher | [1] | ns |
| CDH1 | E-cadherin | higher | [3] | higher in ALI vs. SF |
| IGF-IR | Insulin-like growth factor 1 receptor | higher | [4] | ns |
| FADS1 | fatty acid desaturase 1 | lower | [1] | ns |
| NEFL | Neurofilament, light polypeptide | higher | [1] | ns |
| NID2 | Nidogen 2 | lower | [1] | lower in ALI vs. S and SF |
| BAX | BCL2-associated X protein | lower | [5] | ns |
| Cx43 | Connexin 43 | lower | [1] | ns |
| SPP1 | Secreted phosphoprotein 1 | lower | [6] | ns |
| IGF-II | Insulin-like growth factor | higher | [4] | ns |
| IFNT | Interferon tau | lower | [3, 4]) | ns |
| SLC2A5 | Fructose transporter | higher | [4] | higher in ALI and S vs. SF |
| LIF | Leukemia inhibitory factor | lower | [5] | ns |
| CYP51A1 | Cytochrome P450  family 51, subfamily A, polypeptide 1 | lower | [1] | ns |
| MASH2 | Mammalian achaetescute homologue | higher | [7] | ns |

**References**

1. Clemente M, Lopez-Vidriero I, O'Gaora P, Mehta JP, Forde N, Gutierrez-Adan A, Lonergan P, Rizos D. Transcriptome changes at the initiation of elongation in the bovine conceptus. *Biol Reprod* 2011, 85:285-295.

2. Smith SL, Everts RE, Tian XC, Du F, Sung LY, Rodriguez-Zas SL, Jeong BS, Renard JP, Lewin HA, Yang X. Global gene expression profiles reveal significant nuclear reprogramming by the blastocyst stage after cloning. *Proc Natl Acad Sci U S A* 2005, 102:17582-17587.

3. Wrenzycki C, Herrmann D, Keskintepe L, Martins A, Jr., Sirisathien S, Brackett B, Niemann H. Effects of culture system and protein supplementation on mRNA expression in pre-implantation bovine embryos. *Hum Reprod* 2001, 16:893-901.

4. Lonergan P, Rizos D, Gutierrez-Adan A, Moreira PM, Pintado B, de la Fuente J, Boland MP. Temporal divergence in the pattern of messenger RNA expression in bovine embryos cultured from the zygote to blastocyst stage in vitro or in vivo. *Biol Reprod* 2003, 69:1424-1431.

5. Rizos D, Lonergan P, Boland MP, Arroyo-Garcia R, Pintado B, de la Fuente J, Gutierrez-Adan A. Analysis of differential messenger RNA expression between bovine blastocysts produced in different culture systems: implications for blastocyst quality. *Biol Reprod* 2002, 66:589-595.

6. Kues WA, Sudheer S, Herrmann D, Carnwath JW, Havlicek V, Besenfelder U, Lehrach H, Adjaye J, Niemann H. Genome-wide expression profiling reveals distinct clusters of transcriptional regulation during bovine preimplantation development in vivo. *Proc Natl Acad Sci U S A* 2008, 105:19768-19773.

7. Wrenzycki C, Wells D, Herrmann D, Miller A, Oliver J, Tervit R, Niemann H. Nuclear transfer protocol affects messenger RNA expression patterns in cloned bovine blastocysts. *Biol Reprod* 2001, 65:309-317.
